# The global research of bladder cancer immunotherapy from 2012 to 2021: A bibliometric analysis

**DOI:** 10.3389/fonc.2022.999203

**Published:** 2022-11-14

**Authors:** Qiuqiu Qiu, Can Deng, Hanqiang Li, Junhui Qiu, Zefeng Shen, Yongquan Ding

**Affiliations:** ^1^ Department of Urology, The People’s Hospital of Gaozhou, Maoming, Guangdong, China; ^2^ Department of Urology, Sun Yat-sen Memorial Hospital, Sun Yat-sen University, Guangzhou, Guangdong, China

**Keywords:** bladder cancer, metastatic urothelial carcinoma, immunotherapy, immune checkpoint inhibitor, bibliometrics

## Abstract

Intravesical instillation of Bacillus Calmette-Guérin has been used as an immunotherapy to treat superficial bladder cancer for almost half a century. In recent years, the approval of several monoclonal antibody treatments has transformed the treatment landscape for patients with muscle-invasive or metastatic uroepithelial carcinoma. The purpose of this study was to conduct a thorough review of immunotherapy in bladder cancer through a bibliometric approach. Publications related to bladder cancer immunotherapy were obtained from the Web of Science Core Collection on July 1st, 2022. We conducted a bibliometric analysis of literature information using CiteSpace IV, VOSviewer, and Scimago Graphica, including co-authorship or co-citation of authors, countries/regions, journals, references, and keyword co-occurrence. There was a total of 2,352 papers included, with the most contributions coming from the United States, China, and Italy. The United States had the highest H-index value and was the leading country in this field. Meanwhile, the number of publications in China was steadily growing. The top three productive researchers were Kamat AM, Necchi A, and Shariat SF, with Powles T as the top co-cited author. Most papers were published by the University of Texas System. The majority of papers in this field were published in *Urologic Oncology Seminars and Original Investigations* and *European Urology* was the most influential journal with the highest H-index. The tumor microenvironment and complete molecular characterization may still be the frontier in this research area, allowing us to obtain a better understanding of the pathogenesis and clinical prognosis of bladder cancer. More research are conducted to identify clinically meaningful biomarkers that may provide opportunities for the personalization of bladder cancer therapy. This study provides clinicians and researchers with an overview and helpful guidance on how to choose the research direction and management of bladder cancer immunotherapy.

## Introduction

Bladder cancer (BC) is the 9th most often diagnosed disease in the world, with around 430,000 new cases diagnosed each year, and it has a significant societal impact (1, 2). The incidence of BC in men is higher than that in women (the global sex ratio is 3.5:1) (3). Uroepithelial carcinoma (UC) is the most common type of pathology of BC, and is classified into two types: non-muscle-invasive bladder cancer (NMIBC) and muscle-invasive bladder cancer (MIBC). Approximately 25% of newly diagnosed patients have MIBC or metastatic UC (mUC), which has a poor prognosis (4–6). Moreover, the 5-year overall survival (OS) rate of patient with mUC is 15%, and these patients are no longer candidates for radical surgery (4). Platinum-based combination chemotherapy is the first-line treatment for metastatic UC, with pivotal trials published over 20 years establishing its OS benefit (7). However, there are still some patients who do not benefit from chemotherapy due to advanced age, poor physical condition, or renal insufficiency. Therefore, finding an effevtive treatment for cisplatin-intolerant patients is the focus of most researches.

Immunotherapy has become a focus of attention as a systemic treatment option for many cancers by restarting and maintaining the tumor-immune cycle and restoring the body’s normal anti-tumor immune response. Bacillus Calmette-Guérin (BCG) has been used for decades in a non-muscle invasive setting and was considered the most successful immunotherapy for the treatment of tumors in humans (8). In the last decade, immune checkpoint inhibitors (ICIs) had shown considerable anti-tumor efficacy and drastically changed treatment paradigms for patients with advanced-stage, unresectable, or mUC (9–12). The programmed death receptor 1 (PD-1) and its ligand programmed death receptor ligand 1 (PD-L1) are the most widely studied immune checkpoints for cancer immunotherapy (13, 14). In recent years, the US Food and Drug Administration (FDA) has approved five PD-1/PD-L1 inhibitors for treating patients with advanced UC: atezolizumab, pembrolizumab, nivolumab, durvalumab, and avelumab (15). Significantly, immunotherapy is not suitable for all patients or all kinds of tumors. In fact, the objective response rate (ORR) for the five approved ICIs mentioned earlier was only 13.4-21.1% in Phase II or Phase III trials (8, 16). It is undeniable that the development of immunotherapy, especially ICIs, is of great significance to achieve individualized treatment and improve the OS of patients with metastatic cancer (17). As the number of clinical trials has increased, a large number of papers in BC immunotherapy research have been published in influential journals. However, little research has gone through the process of systematically analyzing and evaluating relevant articles.

Bibliometrics is the study of publishing and communication trends in the spread of information using mathematical approaches (18, 19). By employing various interactive visualization tools and combining information methods, we may be able to visually map the development trend of a specific topic, and simultaneously evaluate the academic literature in this field quantitatively and qualitatively (20, 21). The purpose of our study is to sort out the key contributors and current research status of BC immunotherapy in the past ten years through bibliometric analysis, to provide the research foci and frontiers in this field.

## Materials & methods

The literature searching was conducted online on July 1st, 2022, utilizing the Science Citation Index-Expanded (SCI-E) of the Web of Science Core Collection (WoSCC). WoSCC is one of the most commonly used scientific and technical literature search platforms for bibliometric analysis (22). The following were the searching formula:TS=(Bladder Cancer* OR Bladder Tumor* OR Bladder Neoplasm* OR “cancer near/5 bladder” OR “tumor near/5 bladder”) AND TS=(Immunotherap* OR Immune therap* OR Immunomodulation) AND LA=(English). The papers were published between January 1, 2012 and December 31, 2021, and only original articles and reviews were included.

### Data collection & statistical methods

Two authors independently retrieved the original data from WoSCC and compared the analysis results to ensure the integrity and authenticity of data. The paper records including title, keywords, abstract, author, organization, and reference were all downloaded and preserved in plain text format. From the “Create Citation Report” of WoSCC, the Hirsch index (H-index) (23), total citation and average per item of counties and authors were obtained. Citespace IV (version 6.1.R2) (24)was used to get a dual-map overlay of academic journals, co-citation analysis of references, and keywords with the strongest citation bursts. The VOSviewer software (version 1.6.18) was applied to facilitate the visualization of co-authorship networks and keyword co-occurrence analysis. Besides, we used Scimago Graphica (version 1.0.17) to clearly highlight the cooperation between countries or regions. GraphPad Prism (version 8.0.1) was applied to develop a centered third order polynomial f(x) = ax3 + bx2 + cx + d to estimate the publishing trend of BC immunotherapy literature in 2022 (25). The variable x represents the year, and f(x) represents the number of publications by year.

## Results

### Annual publications and growth forecast

**Figure 1:**
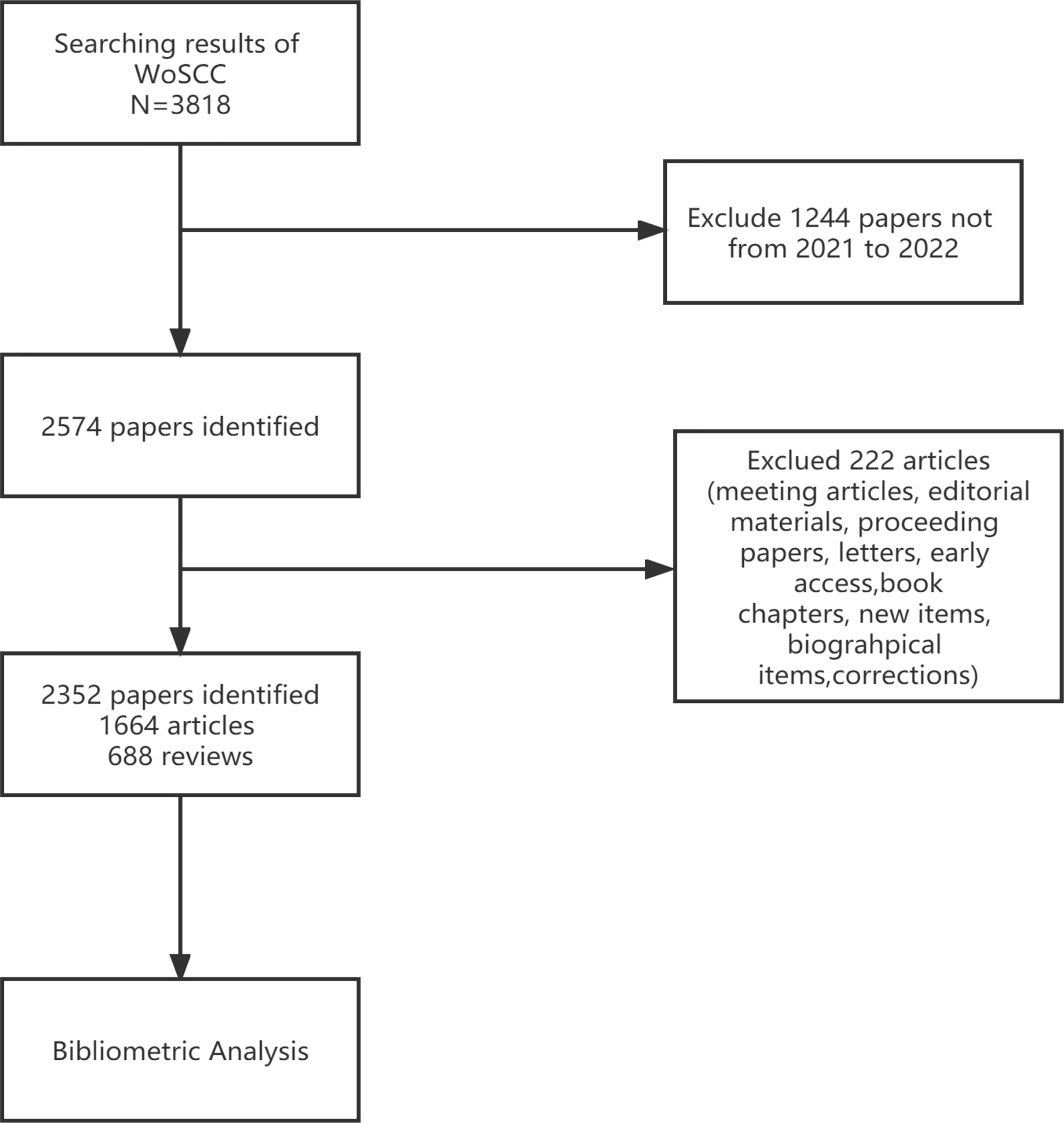
Flow chart of search strategy in this study.

**Figure 2:**
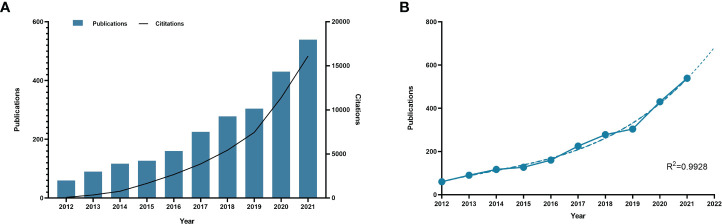
Publication outputs and growth forecast. **(A)** Number of published articles and citations on BC immunotherapy from 2012 to 2021. **(B)** Model-fitted curves of prediction of future publication in BC immunotherapy research.

Through the data retrieval strategy, we found 2,352 papers on BC immunotherapy from WoSCC between 2012 and 2021, with 1,644 original research and 688 reviews (**Figure 1**). All papers had been cited 57,603 times as of the search date, with an H-index of 96 and an average of 24.49 citations per item. The number of BC immunotherapy research papers published in each period climbed steadily, with the number of publications and citations quickly increasing from 2020 (**Figure 2A**). In 2021, there were 539 papers and 16,111 citations, respectively. The polynomial curve fitting of publication growth revealed a significant association between publication year and the number of articles (R^2 =^ 0.9928) (**Figure 2B**). It can be estimated that 606 articles will be published in 2022 using this method.

### Active countries/regions

**Figure 3:**
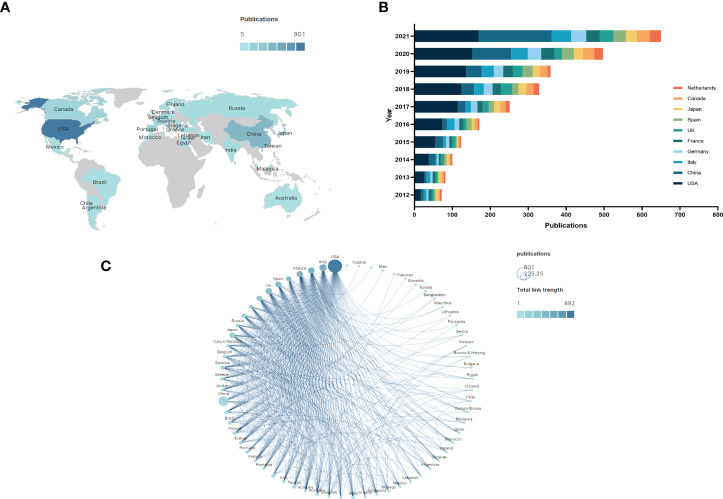
**(A)** The changing trend of the annual publication quantity in the top 10 countries/regions from 2012 to 2021. **(B)** The international collaborations’visualization map of countries/regions.

Papers on BC immunotherapy were published in 68 countries/regions. The global distribution of publications by countries and regions was depicted in **Figure 3A**. The majority of countries and regions across all continents have published a variety of publications, with most of Africa appearing to give less attention to the topic of BC immunotherapy. As seen in **Table 1**, the United States, China, and Italy were the top three productive countries. Approximately 902 papers had been published in the United States, and the H-index and the total number of citations were also among the highest. China published the most in 2021, and it was the only developing country among the top ten contributing countries (**Figure 3B**). Furthermore, we created a visual map of national or regional collaboration using VOSviewer and Scimago Graphica software (**Figure 3C**). The node size denoted the number of publications, while the connection color indicated total link strength (TLS). Obviously, the majority of cooperation was between European and American countries.

**Table 1:** The Top 10 countries/regions that contributed to the publications on BC immunotherapy research.

Rank	Country/region	Count	Percentage (%)	H-index	Total citations	Average citation per paper	TLS
1	USA	902	38.350	81	33,743	37.41	892
2	China	455	19.345	34	6,758	14.85	100
3	Italy	218	9.269	35	5,769	26.46	528
4	Germany	177	7.526	39	8,960	50.62	476
5	France	170	7.228	38	11,768	69.22	467
6	UK	165	7.015	42	12,386	75.07	385
7	Spain	152	6.463	32	8,400	55.26	389
8	Japan	145	6.165	26	3,044	20.99	165
9	Canada	142	6.037	31	3,576	25.18	297
10	Netherlands	108	4.592	32	8,448	78.22	389

TLS, total link strength.

### Active affiliations

**Figure 4:**
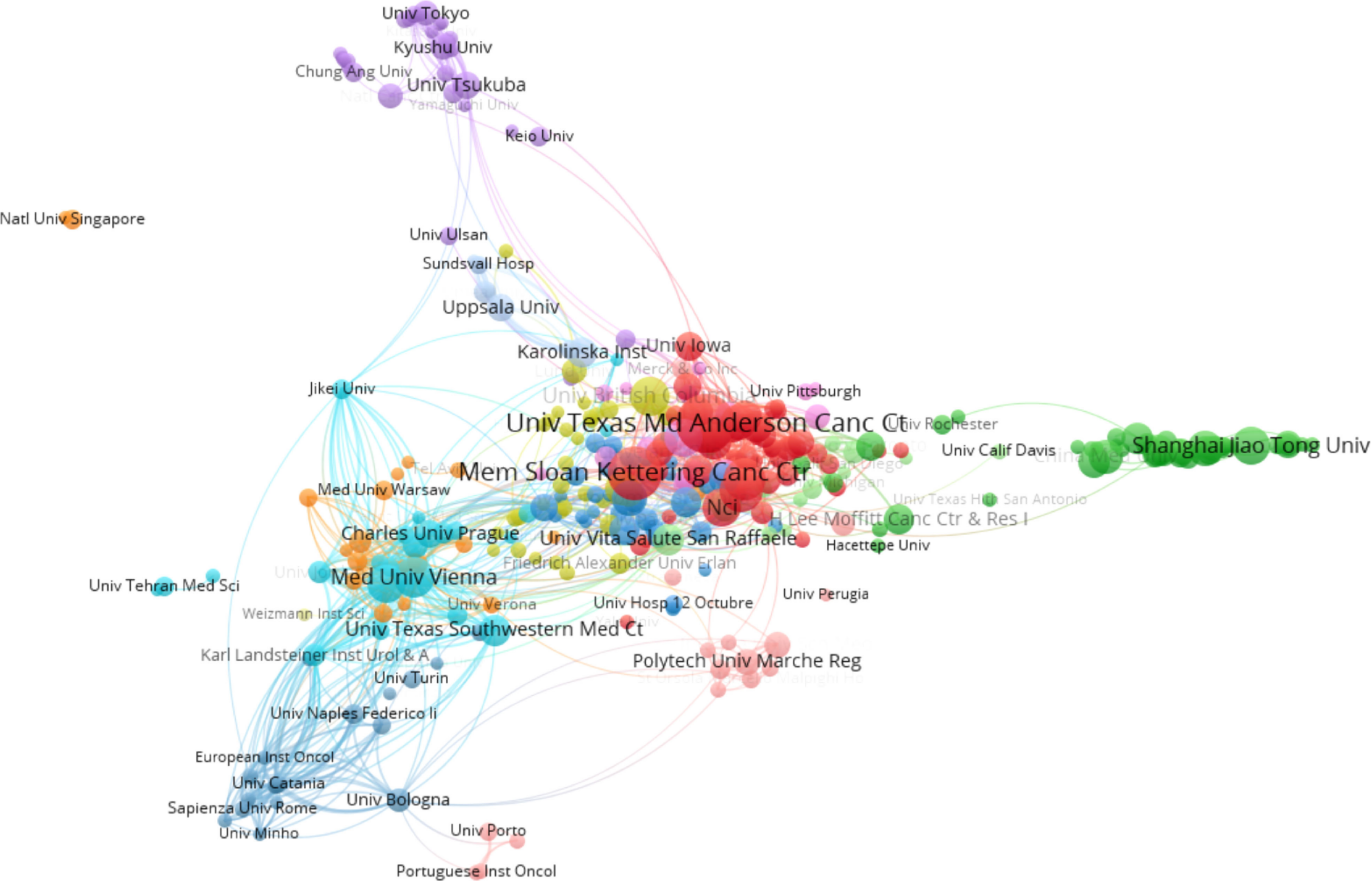
The network map of cooperation between affiliations.

**Table 2** listed the top 10 affiliations that contributed to BC immunotherapy publications. Seven of them were from the United States, with the remaining three coming from France. The top three productive affiliations were the University of Texas System, UTMD Anderson Cancer Center, and Udice French Research Universities. Furthermore, the University of Texas System was the most influential institution in this field with the highest number of publications and H-index. The collaborations between affiliations were not evident, except in the United States (**Figure 4**).

**Table 2:** The Top 10 affiliations that contributed to the publications on BC immunotherapy research.

Rank	Affiliations	Country	Count	Average citation per paper	H-index
1	University of Texas System	USA	174	38.40	42
2	UTMD Anderson Cancer Center	USA	111	35.85	35
3	Udice French Research Universities	France	106	76.63	30
4	Harvard University	USA	100	52.83	29
5	Johns Hopkins University	USA	84	53.15	30
6	University of California System	USA	83	86.34	31
7	Memorial Sloan Kettering Cancer Center	USA	81	71.23	29
8	Assistance Publique Hopitaux Paris	France	66	83.28	23
9	Cornell University	USA	66	29.82	22
10	Unicancer	France	61	69.68	23

### Journals analysis

**Figure 5:**
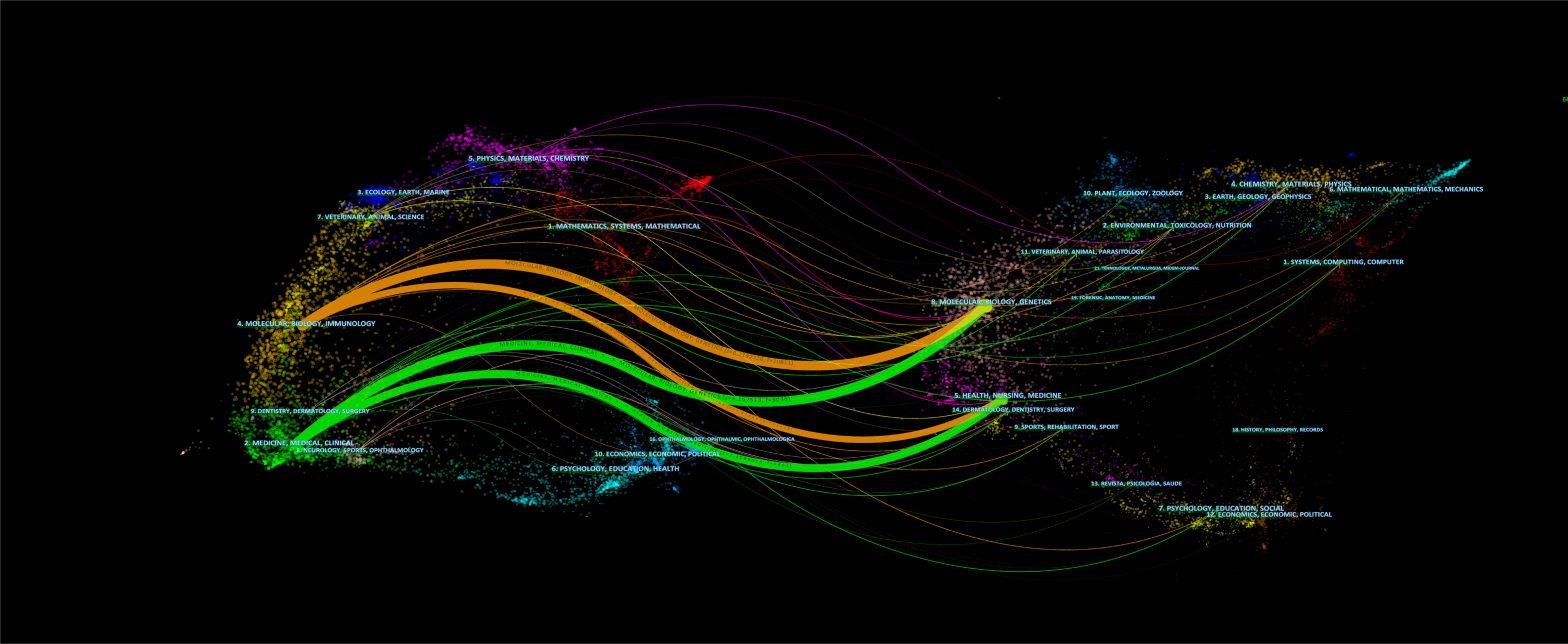
The dual-map overlay of journals.

More than 607 journals had published articles on BC immunotherapy, with the most published in *Urological Oncology Seminars and Original Investigations*, followed by *Frontiers in Oncology* and *European Urology* (**Table 3**). Scientific publications have long been vital tools for scientists and researchers in all sectors to communicate their findings, and the impact factor (IF) of a journal is a crucial aspect in determining its worth and that of included publications (26). Five of the top ten journals were located in Q1, and *European Urology* had the highest IF (**Table 3**). In the dual-map (**Figure 5**), the citing journals and cited journals were on the left and right sides respectively, and each label was centered at the cluster centroid of the relevant journals. As shown in **Figure 5**, there were four primary citation tracks. The papers published in journals of Molecular/Biology/Genetics and Health/Nursing/Medicine were expected to be cited in the majority of papers in Molecular/Biology/Immunology and Medicine/Medical/Clinical journals.

**Table 3:** The Top 10 journals that contributed to the publications on BC immunotherapy research.

Rank	Journal title	Country	Count	H-index	IF 2021	Quartile in category (2021)
1	Urologic Oncology Seminars and Original Investigations	USA	90	18	2.954	Q3
2	Cancers	Cancers	57	10	6.574	Q1
3	Frontiers In Oncology	Switzerland	57	12	5.738	Q2
4	European Urology	Netherlands	52	34	24.267	Q1
5	Clinical Genitourinary Cancer	USA	40	11	3.121	Q2
6	Bladder Cancer	Netherlands	38	7	1.449	Q4
7	World Journal Of Urology	USA	36	11	3.661	Q2
8	Journal Of Urology	USA	35	19	7.600	Q1
9	Oncoimmunology	Netherlands	35	15	7.723	Q1
10	Cancer Immunology Immunotherapy	Netherlands	34	13	6.630	Q1

### Active authors

**Figure 6:**
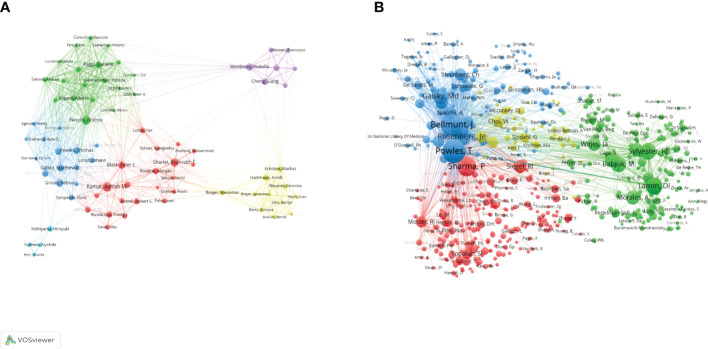
Visualization map of co-authorship **(A)** and co-citation **(B)** analyses of authors generated by VOSviewer software.

This study included 11988 authors in total. **Table 4** showed that the top 10 productive authors contributed 322(13.7%) papers on BC immunotherapy in the last decade. With 42 publications, Kamat AM came in first, followed by Necchi A (27) and Shariat SF (28). They were also among the top three authors with the highest H-index. **Figure 6A** showed the collaborative relationships between authors with at least 10 articles. The size of the nodes represents the number of documents, while the size of the connections between nodes represents the TLS. Co-citation analysis refers to when two documents are simultaneously cited by a third document. Powles T, Bellmunt J, and Sharma P were the top three co-cited authors. We created a visualization map of co-cited authors with VOSviewer (**Figure 6B**). Powles T, the author with the highest TLS, grew and formed stronger collaborative relationships with other authors.

**Table 4:** The Top 10 authors and co-cited authors that contributed to the publications on BC immunotherapy research.

Rank	Author	Contry	Count	H-index	Co-cited Author	Count	TLS
1	Kamat AM	USA	42	19	Powles T	1117	39663
2	Necchi A	USA	39	15	Bellmunt J	1084	37608
3	Shariat SF	Italy	38	15	Sharma P	795	28186
4	Grivas P	USA	32	14	Balar Av	691	25795
5	Powles T	UK	31	20	Rosenberg JE	658	23184
6	Galsky MD	USA	30	14	Sylvester Rj	579	19079
7	Black PC	Canada	29	14	Lamm Dl	563	14513
8	Bellmunt J	USA	28	15	Galsky MD	561	24496
9	Rosenberg JE	USA	28	14	Babjuk M	516	12554
10	Montironi R	Italy	25	11	Kamat AM	508	17512

TLS, total link strength.

### Co-cited references

**Figure 7:**
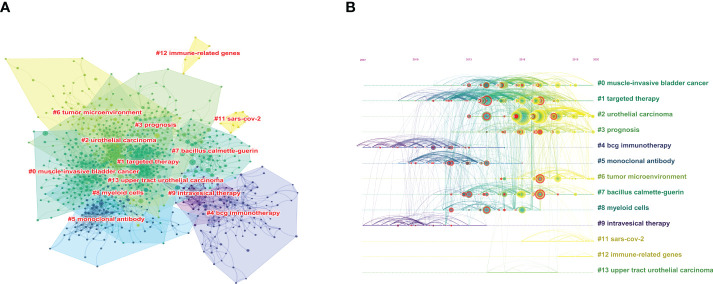
The cluster view map **(A)** and timelines view map **(B)** of co-cited references analysis generated by CiteSpace software.

Co-citation analysis is an effective method for tracking the evolution of scientific disciplines and determining how closely related specialties are to one another (29) Among the top 10 co-cited references in BC immunotherapy research (**Table 5**), 9 articles were published between 2017 and 2018, and cited more than 200 times, with *The Lancet* being the most frequently published journal in the same period. The CiteSpace clustering program was used to conduct a clustering analysis of co-citation references and identify the common subjects of related documents. The **Figure 7A** showed that there were 13 clusters, each of which was made up of several closely related terms. The modularity value (Q-value) was 0.6967 and mean silhouette value (S-value) was 0.8855, suggesting that the clustering structure was considerable and persuasive. Furthermore, as shown in **Figure 7B**, it was obvious that the research focus shifted from #4 BCG immunotherapy, #5 monoclonal antibody, and #9 intravesical therapy to #3 prognosis and #6 tumor microenvironment (TME).

**Table 5:** The Top 10 co-cited references in BC immunotherapy research.

Rank	Co-cited reference	Journal	Author	Year	Citations
1	Atezolizumab in patients with locally advanced and metastatic urothelial carcinoma who have progressed following treatment with platinum-based chemotherapy: a single-arm, multicentre, phase 2 trial	The Lancet	Rosenberg JE et al.	2016	503
2	Pembrolizumab as Second-Line Therapy for Advanced Urothelial Carcinoma	The New England journal of medicine	Bellmunt J et al.	2017	428
3	Atezolizumab as first-line treatment in cisplatin-ineligible patients with locally advanced and metastatic urothelial carcinoma: a single-arm, multicentre, phase 2 trial	The Lancet	Balar AV et al.	2017	316
4	Nivolumab in metastatic urothelial carcinoma after platinum therapy (CheckMate 275): a multicentre, single-arm, phase 2 trial	Lancet Oncology	Sharma P et al.	2017	306
5	MPDL3280A (anti-PD-L1) treatment leads to clinical activity in metastatic bladder cancer	Nature	Powles T et al.	2014	281
6	Atezolizumab versus chemotherapy in patients with platinum-treated locally advanced or metastatic urothelial carcinoma (IMvigor211): a multicentre, open-label, phase 3 randomised controlled trial	The Lancet	Powles T et al.	2018	261
7	EAU Guidelines on Non-Muscle-invasive Urothelial Carcinoma of the Bladder: Update 2016	European Urology	Babjuk M et al.	2017	258
8	Safety and Efficacy of Durvalumab (MEDI4736), an Anti-Programmed Cell Death Ligand-1 Immune Checkpoint Inhibitor, in Patients With Advanced Urothelial Bladder Cancer	Journal of Clinical Oncology	Massard C et al.	2016	208
9	First-line pembrolizumab in cisplatin-ineligible patients with locally advanced and unresectable or metastatic urothelial cancer (KEYNOTE-052): a multicentre, single-arm, phase 2 study	The Lancet	Balar AV et al.	2017	205
10	EAU Guidelines on Non-Muscle-invasive Urothelial Carcinoma of the Bladder: Update 2016	Cell	Robertson AG et al.	2017	191

### Keywords co-occurrences

**Figure 8:**
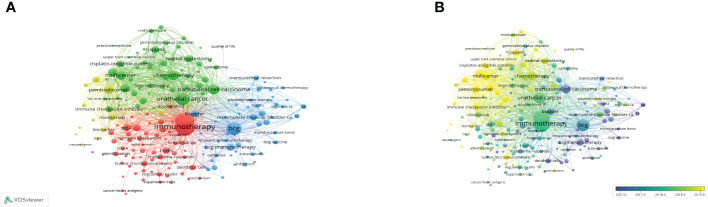
The network visualization map **(A)** and overlay visualization map **(B)** of the 114 phrases with at least 10 times frequency were created by VOSviewer. The more frequently the keywords appear, the larger the nodes is.

By examining the co-occurrence of word pairs or noun phrases in the collection, keywords co-occurrence analysis helps to evaluate the relationship between themes in the discipline represented by the collection. After deleting meaningless words and merging consent words, 114 keywords were visualized through VOSviewer. Keywords were primarily grouped into four clusters and as indicated in **Figure 8A**, the keywords in red cluster were primarily concerned with the classification and mechanism of BC immunotherapy, while the keywords in green cluster primarily studied chemotherapy in UC and the keywords in blue cluster mainly focus on BCG immunotherapy. Furthermore, the dispersive yellow clusters reflect clinical trial papers for ICIs. The chronological order of keywords was represented by different colors of nodes in the overlay visualization map (**Figure 8B**). Keywords like “transitional cell carcinoma”, “BCG immunotherapy”, “intravesical immunotherapy” and “dendritic cells” were prominent in the early phases. Besides, “pembrolizumab”, “ICI”, “multicenter”, “cisplatin” and “cisplatin-ineligible patients” appeared very often in the last two years and could still be the foci of future research.

### Keywords with citation burst

**Figure 9:**
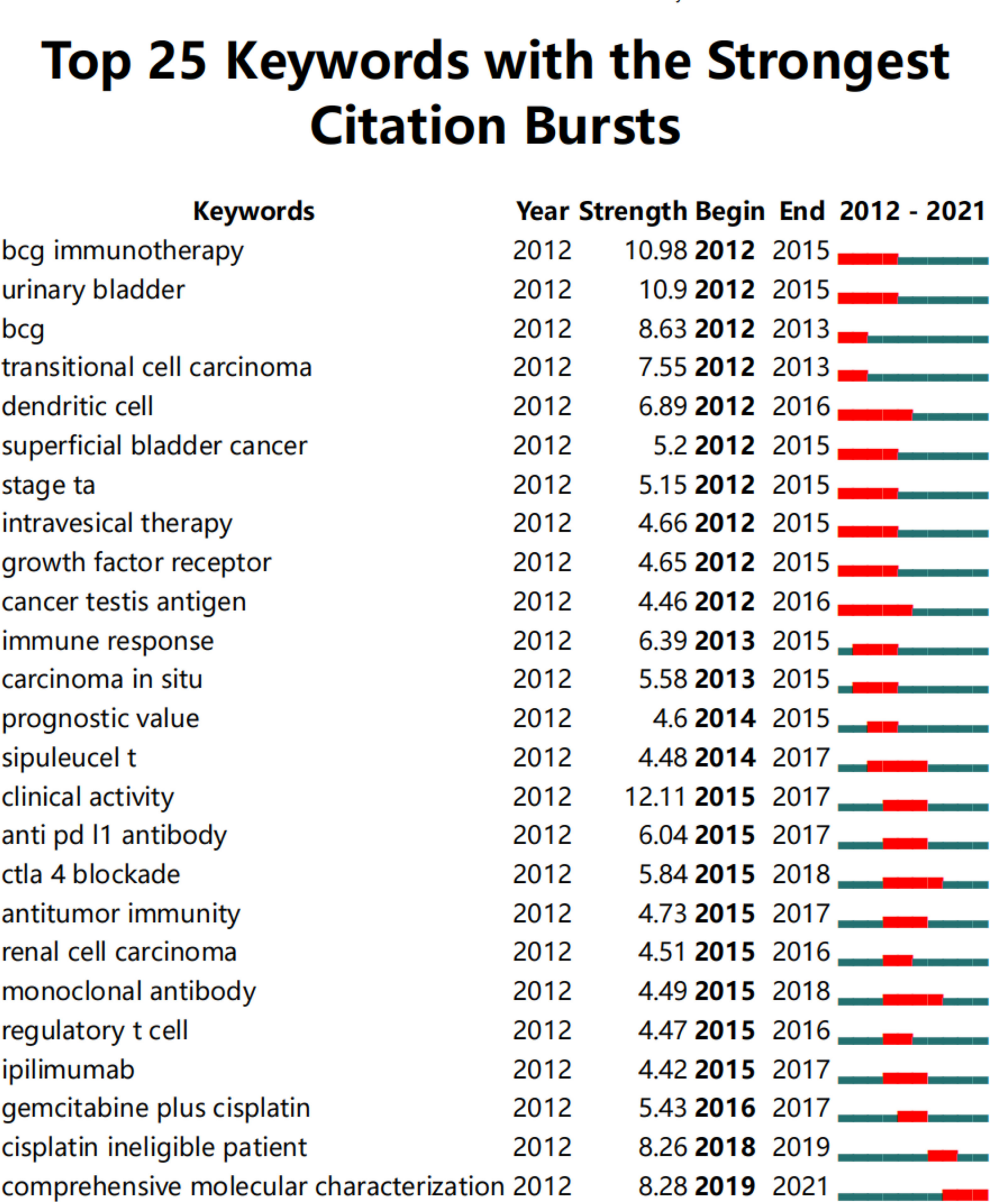
The top 25 keywords with the highest citation bursts in BC immunotherapy (generated by CiteSpace software).

The top 25 keywords with the highest citation burst of BC immunotherapy were listed in **Figure 9**. Studying the recent emergent keywords can accurately grasp the frontier of subject research. With a burst strength of more than 10, the terms “BCG immunotherapy” and “clinical activity” indicated the research hotspot of BC immunotherapy in the last ten years. “Dendritic cells” and “cancer testing antigen” were the keywords that lasted the longest, from 2012 to 2016. “Comprehensive molecular characterization (CMC)” is the only keyword with citation burst up till now.

## Discussion

### General information

With the advent of the era of fast-growing tumor immunotherapy, we have witnessed the approval of multiple ICIs for BC in the past few years, bringing new treatment options for patients with advanced BC. Following BCG intravesical infusion therapy, this is another significant advance in immunotherapy for BC. There have been a significant number of publications in this field after years of research. With the most publications and the highest H-index, the United States was the country that made the biggest contribution in this field. As shown in **Figure 3B**, China has already surpassed the United States in terms of publications in 2021 and will become one of the major research forces in the coming years. However, China has the lowest TLS among the top ten productive countries and needs to strengthen its research cooperation with other countries to produce higher-quality articles. The University of Texas System, UTMD Anderson Cancer Center, and Udice French Research Universities were the most active affiliations in BC immunotherapy research. Certainly, *European Urology* was the most influential journal with the highest IF and H-index.

Kamat AM, from UTMD, had published several studies on BCG immunotherapy, with a focus on the best treatment schedule for BCG and tools for accurately predicting patient response (2, 30, 31). The preliminary efficacy of PD-L1 (atezolizumab) was reported by Powles T et al. (32) in 2014. In the same year, FDA approved atezolizumab as a breakthrough therapy based on this research. Bellmunt J meticulously assessed the results of ICIs trials, paying special focus to mechanisms of resistance and biomarkers of immunotherapy response (4, 33–35). Meanwhile, Sharma P worked on the efficacy and safety of anti-CTLA-4 immunotherapy (36, 37). They were all from the United States and had made major contributions to the research of BC immunotherapy.

### Current subtopics

#### BCG immunotherapy

In the early 1970s, Morales et al. (38) first reported the use of BCG in the bladder could effectively treat and prevent tumors. As an immunotherapy for NMIBC, BCG intravesical instillation has been shown to reduce postoperative recurrence and progression (27, 28, 39). The National Comprehensive Cancer Network (NCNN) recommends intravesical BCG for high-grade Ta, all T1, and any Tis tumors, citing Category 1 results (40). However, the exact mechanism of BCG in the treatment of bladder tumors is still unknown. An increase in macrophages in the TME, the urinary bladder wall around the tumor, and urine was believed to be responsible for protective action of BCG (41, 42). The current study (40, 43, 44) found that trained immunity was one of the key mechanisms of BCG immunotherapy and might provide protection against COVID-19, which may explain why “SARS-CoV-2” may be a potential research direction from **Figure 7B**. Furthermore, the possibility of combining BCG with other therapies, such as ICIs (45, 46), is gaining increasing interest, and more researches are required to develop new strategies.

#### Immune checkpoint inhibitors

In 2015, ICIs emerged as a hot research topic in the field (**Figure 9**). In **Table 5**, the top 10 co-cited references mainly demonstrated that ICIs had a clinically confirmed therapeutic effect. Rosenberg JE et al. (47) published the results of imvigor-211 in *The Lancet* in 2016, including 310 patients with urothelial carcinoma who were resistant to first-line platinum chemotherapy and were treated with atezolizumab alone. In his study, the primary endpoint had an ORR of 15%, with a CRR of 5%. On May 18, 2016, the FDA approved atezolizumab for the second-line treatment of platinum-resistant urothelial carcinoma based on the research findings. Furthermore, based on the study of Balar AV et al. (48), atezolizumab was approved as the first-line treatment in 2017. Pembrolizumab was connected to a three-month increase in OS when compared to chemotherapy, according to the study by Bellmunt J et al. (49). In addition to single-agent immunotherapy, multiple clinical trials had evaluated the efficacy and safety of PD-1/PD-L1 inhibitors combined with chemotherapy or CTLA-4 inhibitors. A phase 2 trial of ipilimumab in combination with cisplatin plus gemcitabine was completed in 2016, opening the way to further combination therapy (2, 50). However, in 2020, three phase-III trials (IMvigor130 (51), KEYNOTE361 (52), DANUBE (53)) found insufficient OS improvements and did not support the use of PD-1/L1 combined chemotherapy or immunotherapy combination as first-line treatment for platinum-eligible patients. Besides, very few studies investigated immunotherapy in combination with radiation or targeted therapy. Further basic and clinical research efforts are needed to determine the optimal therapy with ICIs for BC.

### Research frontiers

Based on the analysis of co-cited references, researchers have recently shown an increased interest in TME. The TME includes the blood vessels, immune cells, fibroblasts, bone marrow-derived inflammatory cells, numerous signaling chemicals, and the extracellular matrix that surrounds tumor cells (54, 55). Even though immunotherapy has an anti-tumor effect on the TME, the majority of patients still fail to respond to ICIs. According to multiple studies, first-line response rates for ICIs drugs are less than 25%, with second-line response rates ranging from 15% to 20% without predictive biomarkers (48, 55–61). More recent research had concentrated on markers for predicting ICIs response. In the IMvigor 210 phase II research, the ORR in the high PD-L1 expression group was 26%, while it was only 8% in the no PD-L1 expression group (47). However, as a predictive biomarker, PD-L1 had some limitations, as it was predictive in only 28.9% of FDA-approved drugs (14). According to the results of The PURE-01, they found a correlation between high tumor mutational burden (TMB) and immunotherapy response, but there was no such relationship with atezolizumab in the light of the ABACUS research findings (7, 62, 63). As a result, pursuing molecular markers testing for clinical decision-making must be approached with caution.

The CMC is another important research frontier in the field of BC immunotherapy (**Figure 9**). Improved sequencing technologies have contributed in the rapid molecular characterization of UC, allowing us to gain a better knowledge of the disease’s pathophysiology (64, 65). Aurélie Kamoun et al. (66) identified six MIBC molecular subtypes: luminal papillary (LumP), luminalnon specified (LumU), luminal unstable (LumNS), stroma-rich, basal/squamous (Ba/Sq) and neuroendocrine-like (NE-like). It had been reported that LumP tumors had a better prognosis than Ba/Sq tumors. FGFR3 mutations were found in abundance in LumP tumors. Furthermore, Erdafitinib, a pan-FGFR inhibitor, was the first FDA-approved targeted therapy for metastatic UC with susceptible FGFR2/3 alterations following platinum-containing chemotherapy (64, 67). Enfortumab vendotin (an antibody-drug) is another breakthrough therapy for metastatic UC patients who have previously received ICIs (4, 68–70). Besides, mutations in carcinogenic pathways, such as the RTK–MAPK and PI3K–MTOR pathways, have been revealed to be helpful as therapeutic targets in bladder cancer by the Cancer Genome Atlas (TCGA) project (71, 72). A deeper understanding of the underlying molecular characterizations and carcinogenic pathways could lead to the identification of prognostic and predictive markers and the development of personalized cancer therapy of BC (73–75).

## Limitations

There were certain limitations in our study. Firstly, the articles in the study were only obtained from the SCI-E of the WoSCC. We disregard other huge databases and some quality researches may be omitting. Secondly, some important previous or current studies may have been ignored because our investigation focused on the development and research hotspots of immunotherapy for BC between 2012 and 2021. Thirdly, only English-language papers were chosen from the database, resulting in an inadequate analysis.

## Conclusion

As far as we know, this is the first comprehensive bibliometric analysis of developmental trends in BC immunotherapy based on related papers over the last decade. The papers in this field still show a rising trend, and the focus of related research has shifted from BCG immunotherapy to ICIs. Anti-PD-1/PDL-1 antibodies have shown certain efficacy and safety in advanced BC in most preliminary clinical trials. More effort has been focused to explore potential biomarkers, patient selection, and combined therapeutic techniques of ICIs. The tumor microenvironment and comprehensive molecular characterization may still be the hotspots in this field and these findings are beginning to change the treatment landscape of BC therapy. More research are needed to identify validated predictive molecular markers for ICIs reactions, which will contribute to the development of BC precision medicine. We also analyzed the countries, institutions, magazines, and authors that contributed the most to this research field. Our findings may help researchers have a general overview of the landscape and current trends in BC immunotherapy.

## Data availability statement

The datasets presented in this study can be found in online repositories. The names of the repository/repositories and accession number(s) can be found below: https://www.webofscience.com/wos/alldb/basic-search.

## Author contributions

YD and ZS conceived the study. QQ and CD collected the data and wrote the manuscript. HL and JQ analyzed the data. YD, ZS and QQ revised and reviewed the manuscript All authors contributed to the article and approved the submitted version.

## Acknowledgments

The authors thank Dr. Weibo Zhong and Dr. Yukun Wu for their effort in polishing the English content of this manuscript.

## Conflict of interest

The authors declare that the research was conducted in the absence of any commercial or financial relationships that could be construed as a potential conflict of interest.

## Publisher’s note

All claims expressed in this article are solely those of the authors and do not necessarily represent those of their affiliated organizations, or those of the publisher, the editors and the reviewers. Any product that may be evaluated in this article, or claim that may be made by its manufacturer, is not guaranteed or endorsed by the publisher.
